# Expanding the Phenotypic Spectrum of SPG4: Autism Spectrum Disorder in Early-Onset and Complex SPAST-HSP and Case Study

**DOI:** 10.3390/genes16080970

**Published:** 2025-08-18

**Authors:** Carlo Alberto Quaranta, Alice Gardani, Giulia Andorno, Anna Pichiecchio, Simone Gana, Renato Borgatti, Simona Orcesi

**Affiliations:** 1 Department of Brain and Behavioral Sciences, University of Pavia, 27100 Pavia, Italy; carloalberto.quaranta01@universitadipavia.it (C.A.Q.); giulia.andorno01@universitadipavia.it (G.A.); anna.pichiecchio@mondino.it (A.P.); renato.borgatti@mondino.it (R.B.); 2 Department of Child Neurology and Psychiatry, IRCCS Mondino Foundation, 27100 Pavia, Italy; alice.gardani@mondino.it; 3 Neuroradiology Department, IRCCS Mondino Foundation, 27100 Pavia, Italy; 4 Neurogenetics Research Center, IRCCS Mondino Foundation, 27100 Pavia, Italy; simone.gana@mondino.it

**Keywords:** autism spectrum disorder, SPAST, neurodevelopmental disorder, hereditary spastic paraplegia, early-onset

## Abstract

Background/Objectives: Hereditary spastic paraplegias (HSPs) comprise a heterogenous spectrum of rare neurogenetic disorders predominantly characterized by progressive spasticity and weakness of the lower extremities. Among autosomal-dominant forms of HSP, molecular defects in the *SPAST* gene—particularly those associated with the SPG4 subtype—represent the most frequent genetic cause. SPAST encodes spastin, a microtubule-severing ATPase, crucial for cytoskeletal remodeling, neuronal connectivity, and intracellular trafficking. Disruption of spastin function can impair neurite outgrowth and synaptic formation, processes increasingly implicated in neurodevelopmental disorders (NDDs). Methods: We conducted a comprehensive clinical, neurological, and dysmorphological evaluation of a 4-year-old male. Standardized neuropsychological assessments were administered. Whole-exome sequencing (WES) was performed to identify underlying genetic causes. EEG and 3T-brain MRI were also acquired. Results: The proband harbored two novel de novo heterozygous missense variants in cis of the SPAST gene, displaying the typical features of early-onset and complex HSP, in addition to global developmental delay and severe autism spectrum disorder (ASD), an underexplored manifestation in this rare genetic disorder. Conclusions: These findings broaden the clinical and mutational spectrum of SPG4, underscoring the importance of considering SPAST gene analysis in patients with ASD in the early years of life and early motor delay, even in the presence of only subtle pyramidal signs. We advocate for comprehensive neuropsychiatric assessment in the diagnostic pathway of early-onset complex HSP presentations and support further investigation into the role of spastin in neuronal connectivity.

## 1. Introduction

Hereditary spastic paraplegias (HSPs), formerly known as Strümpell–Lorrain disease, are a heterogeneous and rare group of genetic disorders. They were first described in 1880 by Adolf von Strümpell, who reported two adult brothers of Estonian origin presenting with “primitive combined sclerosis of the pyramidal tracts, spinocerebellar tracts, and Goll’s tracts.” Throughout the 19th century, further clinical descriptions emerged primarily in France, England, and Germany. Early accounts emphasized an autosomal-dominant pattern of inheritance, onset in young adulthood, and selective dysfunction of the corticospinal tracts, resulting in progressive spasticity and weakness of the lower limbs. Cognitive function was typically reported as preserved, and HSP was historically regarded as a “pure” motor disorder. Over time, however, the identification of “complex” forms, accompanied by additional neurological or systemic features, has broadened the phenotypic spectrum of these conditions [1,2]. These disorders are, now, classified into two main categories: ‘pure’ HSP, where spastic paraplegia is the core clinical feature, and ‘complex’ HSP, which is associated with additional neurological symptoms.

Among the many genetic causes of HSP, pathogenic variants in the SPAST gene, which encodes spastin, a microtubule-severing ATPase located on chromosome 2p22.3, are the most common [3]. HSP-SPAST (also known as SPG4) accounts for approximately 30–80% of all autosomal-dominant cases of HSP [4]. SPAST variants are associated with a wide range of clinical presentations, from the classical pure form of spastic paraplegia to more complex, severe phenotypes. Studies have shown that the majority of HSP-SPAST cases are inherited, with considerable variability in age of onset and symptom severity within families and across generations [5].

A particularly noteworthy aspect of SPAST-related HSP is the occurrence of rare de novo pathogenic variants, which can result in more severe and early-onset forms of the disorder. Recent studies, including those by Mo et al. and Schieving et al., have identified and thoroughly described cases of de novo HSP-SPAST leading to early-onset spastic paraplegia, accompanied by other motor disorders, progressive cognitive decline, motor disorders, and bulbar dysfunction—features typically absent in familial cases with the same SPAST variants [6,7,8,9]. These severe manifestations underscore the clinical heterogeneity of SPAST molecular defects and suggest that patients with de novo pathogenic variants may experience a more aggressive disease course [10,11].

Gene expression and functional studies of SPAST also indicate its vital role in early neurodevelopment and neural integrity. Notably, SPAST expression begins during early embryonic stages, where it is actively transcribed in neural stem cells (NSCs) and perinatal mouse brain tissues [12]. In SPAST-deficient murine models, researchers observed impaired NSC proliferation and diminished neuronal lineage differentiation, linked to disrupted microtubule dynamics within primary cilia [13]. These findings suggest that spastin is essential not only for maintaining neural architecture but also for supporting progenitor cell proliferation and neuronal differentiation.

Further integrative studies emphasize spastin’s crucial involvement in axonal and dendritic development. As a microtubule-severing ATPase, spastin regulates cytoskeletal remodeling, axonal branching, transport, and regeneration, processes fundamental to proper neuronal connectivity [12,13,14,15]. Loss of function or reduced spastin levels lead to axonal swellings, disrupted transport, and defective neurite maintenance in both in vitro and in vivo models [16,17,18].

Taken together, these molecular and cellular expression data support the notion that SPAST plays a broader neurodevelopmental role than previously recognized, extending beyond pure motor neuron maintenance to include regulation of neural progenitor proliferation and synaptic connectivity—pathways increasingly implicated in neurodevelopmental disorders such as autism spectrum disorder (ASD).

We present a patient with a clinical and genetic diagnosis of early-onset and severe complex HSP-SPAST who exhibits a complex neurodevelopmental disorder with severe autism spectrum disorder (ASD) as classified by the DSM-5-TR, evident before the appearance of pyramidal signs in the lower limbs, expanding the spectrum of SPG4.

## 2. Materials and Methods

A clinical, neurological, and dysmorphological evaluation was conducted by a multidisciplinary team of neuropsychiatrists (S.O., C.A.Q., G.A.), a neuropsychomotor therapist (A.G.), a clinical genetist (S.G.), and a neuroradiologist (A.P.). Moreover, a specific age-related neuropsychological assessment battery (Autism Diagnostic Observation Schedule [ADOS] 2 Toddler Module, Autism Diagnostic Interview [ADI-R], Griffith Mental Development Scale [GMDS], and Vineland Adaptive Behavior Scale II [VABS-II]) was administered at 2 and 3 years of age.

Written informed consent was obtained from the patient’s legal guardians for the use of clinical and instrumental data for research, educational, and publication purposes. Ethical approval from the institutional review board was not required, as all procedures were performed as part of routine clinical diagnostic assessment.

Neuroimaging: The patient underwent a 3T brain MRI with sedation using multiplanar T1- and T2-weighted images with age-appropriate TR and TE values.

EEG: Digital scalp video-EEG recordings in wakefulness and during daytime sleep were obtained using a preformed cap with electrodes placed according to the international 10–20 system with a standard 19-channel NicoletOne EEG system.

Chromosomal Microarray (CMA): The whole-genomic screening of chromosomal rearrangements was performed by array-comparative genomic hybridization (CGH) using the SurePrint G3 Human CGH 4 × 180K kit (Agilent Technologies, Santa Clara, CA, USA), according to the manufacturers’ recommendations. Data analysis was carried out with the Agilent Cytogenomics v.5.2.0.20 software. All nucleotide positions refer to the GRCh37/hg19 human genome assembly. Whenever possible, segregation analysis was performed on parental DNA samples. CNVs of interest were searched in the DECIPHER (https://www.deciphergenomics.org/, accessed on 15 June 2025) and DGV (https://dgv.tcag.ca/, accessed on 15 June 2025) databases. CNVs were classified according to the ACMG/ClinGen guidelines [19]. Only pathogenic or likely pathogenic CNVs associated with the reported proband’s phenotype were taken into account.

FMR1 Testing: FMR1 testing was performed using the Amplidex kit PCR/CE FMR1 (Asuragen, Austin, TX, USA) following the manufacturer’s recommendations.

Whole-Exome Sequencing: Proband-only whole-exome sequencing (WES) was performed (Twist Human Core Exome Kit, Twist Bioscience, South San Francisco, CA, USA) on a NovasSeq6000 platform (Illumina, San Diego, CA, USA). Bioinformatics analysis was carried out by aligning sequences to the human reference genome (GRCh37; http://genome.ucsc.edu, accessed on 15 June 2025) using BWA v0.7.5 [20]. Variants were called with the GATK Unified Genotyper [21] and annotated through the eVANT v1.3 software (enGenome, Pavia, Italy; https://www.engenome.com, accessed on 15 June 2025). Variant filtering was based on quality/coverage depth (≥15) and minor allele frequency (MAF  <  0.01) as reported in the Genome Aggregation Database (gnomAD) [22]. Variants were further filtered to include, according with a phenotype-driven approach, only coding, splicing, and 5 prime UTR premature start codon gain variants, excluding deep non-coding variants and synonymous variants with no predicted impact on the RNA splicing process. Variants of interest were evaluated in silico by considering several predictors, including Deleterious Annotation of genetic variants using Neural Networks (DANN) [23], Combined Annotation-Dependent Depletion (CADD) [24], Polymorphism Phenotyping v2 (PolyPhen-2) [25], Sorting Intolerant from Tolerant (SIFT) [26], pseudo amino acid composition to score human protein-coding variants (PaPI) [27], and Mutation Taster [28]. Finally, variants were classified according to the latest ACMG criteria [29,30]. All candidate or pathogenic variants were verified by Sanger sequencing. Targeted Sanger sequencing was also performed in parental samples to determine compound heterozygosity and to establish the origin of the identified causative variants. Finally, exome data underwent a coverage-based analysis of copy number variants (CNVs) by the eVai platform (enGenome, Pavia, Italy; https://www.engenome.com, accessed on 15 June 2025), which combines the ExomeDepth and EXCAVATOR pipelines [31,32]. WES-based CNV calling, in our patient, excluded any relevant genomic imbalance.

## 3. Results

### 3.1. Clinical Assessment

The proband, a 4-year-old boy, is the first child of healthy, unrelated European parents with no significant medical history. The pregnancy was uneventful, with no reported exposure to tobacco, alcohol, illicit substances, or prescription medications during pregnancy, except for a threatened miscarriage in the first trimester, managed with progesterone. Born at 40 + 1 weeks, LGA, he had a length of 54 cm (99th percentile), weight of 3955 g (92nd percentile), and head circumference of 35 cm (62nd percentile). Apgar scores at 1, 5, and 10 min were 7, 8, and 9, respectively. Positive pressure ventilation was needed for 20 min, with gradual improvement. No feeding issues were noted, except for selective eating, characterized by a strong preference for soft textures and a limited range of accepted foods, with categorical refusal of other food types regardless of nutritional value or exposure

At 2 years, neurodevelopmental milestones were delayed, notably in language (limited vocalizations) and motor skills (gross/fine). Structured assessments at 2 years of age (ADOS 2 Toddler Module and ADI-R) revealed impaired reciprocal interactions, limited eye contact, and stereotyped, repetitive use of objects, suggesting moderate-to-severe ASD risk. There was also an absence of pretend, imitative, or symbolic play and limited social engagement.

**Figure 1:**
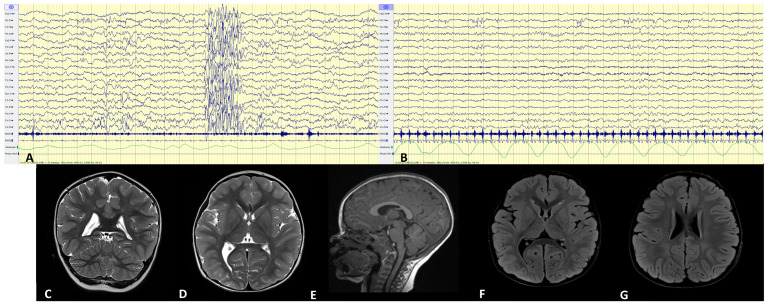
Sleep EEGs show, during drowsiness, (**A**) generalized discharges of slow, sharp, high-voltage waves and spike waves, with no clinical correlate; during wakefulness and sleep, (**B**) spike waves in the right fronto-temporal regions, which increase during drowsiness and sleep. All EEGs are shown at filter settings: high pass of 0.5 Hz, low pass of 40 Hz, notch of 50 Hz, and time base of 15 mm/sec. The sensitivity is 200 μV/cm (**A**,**B**). Imaging studies with MRI at the age of 3 years (**C**–**G**). Coronal (**C**) and axial (**D**) T2-weighted images show a normal ventricular system with moderate asymmetry of the lateral ventricles, particularly in the posterior portions, with the right side being larger than the left. Sagittal (**E**) T1-weighted image shows a normal cerebellum and corpus callosum. Axial (**F**,**G**) FLAIR images show slight hyperintensity affecting the deep superotrigonal white matter bilaterally, not pathological and related to the presence of dilated perivascular spaces; previously described ventricular asymmetry is also evident.

Brain MRI showed no significant abnormalities, except for faint hyperintensities in the parietal periventricular white matter and mild right-sided ventricular asymmetry. Sleep-EEG revealed spike-and-wave abnormalities in the right fronto-temporal regions and generalized slow-wave discharges with paroxysmal activity, without clinical correlates (see Figure 1). The proband has never experienced clinical seizures and he was not taking any prescription medications.

At 3 years of age, neuropsychological and developmental assessments confirmed a diagnosis of ASD and global developmental delay. GMDS indicated a low global developmental level (GQ < 20, <1st percentile), confirmed by VABS-II, which revealed markedly low adaptive functioning (Deviation IQ: 27; <0.1st percentile).

The child has held up his head (since 5 months), sat unsupported (since 7 months) and stood (since 11 months), but he was unable to walk without assistance, and expressive language was absent. He presented pyramidal signs, including lower limb spasticity, hyperreflexia (polykinetic patellar reflexes), clonus at the right Achilles tendon, and bilateral Babinski signs. Disease severity, assessed using the Spastic Paraplegia Rating Scale (SPRS), was scored at 31. ADI-R and ADOS-2 highlighted significant social deficits and repetitive, stereotyped behaviors.

**Figure 2:**
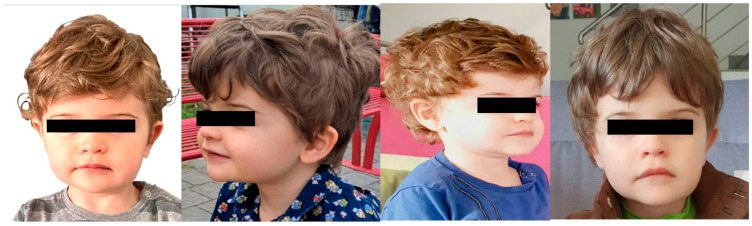
Proband at age 3 years old showing square-shaped face, thin eyebrows with sparse medial thirds, hypertelorism, mild down-slanting features, Cupid’s bow-shaped upper lip, and mild retrognathia.

Physical examination revealed a square-shaped face, thin eyebrows with sparse medial thirds, hypertelorism, mild down-slanting eyelids, Cupid’s bow-shaped upper lip, and mild retrognathia (see Figure 2).

Audiological evaluation and auditory evoked potential, neuro-ophtalmologic evaluation and visual evoked potentials, echocardiogram, electrocardiogram—Holter, abdominal ultrasound, and metabolic screening, comprehensive of plasma and urinary amino acids and organic acids, provided results within the normal limits.

### 3.2. Molecular Genetics

Array-CGH excluded any relevant genomic imbalance; FMR1 testing gave a negative result. Proband-only WES analysis, after variant filtering and prioritization, disclosed two novel de novo heterozygous missense variants in cis of the SPAST gene (NM_014946.4), classified as likely pathogenic: c.1330G>T (p.Asp444Tyr) and c.1333A>T (p.Ser445Cys), leading to a diagnosis of SPG4 disorder. Segregation analysis by Sanger sequencing failed to detect the variants in both parents. No other potentially relevant variants were identified in more than 2000 developmental disorder-related genes (DDG2P panel), in more than 100 spastic paraplegia-related genes, in more than 400 metabolic disorder-related genes, and in almost 500 mitochondrial disorder-related genes, as listed in PanelApp [33] (https://panelapp.genomicsengland.co.uk/, accessed on 15 June 2025). WES-based CNV calling excluded any relevant genomic imbalance.

## 4. Discussion

The phenotypic spectrum of early-onset complex forms of SPAST-HSP appears broad and heterogeneous. All patients reported in the literature to date presented with motor developmental delay associated variably with progressive spasticity of the limbs, extrapyramidal movement disorders, intellectual disability or cognitive decline, epilepsy, neurogenic bladder dysfunction, and gastrointestinal dysmotility [6,7,8,9].

In our patient, the earliest manifestations at 24 months included global developmental delay and severe autistic features—such as pronounced stereotypies, restricted and repetitive behaviors, and impaired reciprocal social interaction—which ultimately led to a diagnosis of level 3 autism spectrum disorder (ASD), using accurate and standardized tools according to the DSM-5-TR criteria. Although our patient initially lacked pyramidal signs, he later developed the classical features of early-onset and complex SPG4, including lower-limb spasticity and clinical signs of corticospinal tract involvement. The severity of his motor phenotype, quantified using validated scales, was consistent with that reported in the literature for early-onset cases. He also presented additional neurological features including global developmental delay, non-verbal communication, and EEG abnormalities, although no bladder or gastrointestinal symptoms were observed.

This case is particularly noteworthy given the scarcity of literature linking pathogenic SPAST variants to ASD. To date, ASD has been reported in only five patients harboring SPAST molecular defects. One article described three individuals with two truncating and one missense variant, all presenting with ASD; however, comprehensive neuropsychiatric characterization, familial history, and genetic analyses were lacking, limiting interpretation and the exclusion of dual diagnoses [34]. Another report described a patient with a mosaic SPAST mutation and a concurrent 1q21.1 copy number variant (CNV), a genomic region strongly associated with neurodevelopmental disorders. As the authors of that study concluded, the 1q21.1 CNV is the more likely driver of ASD, while the SPAST variant likely accounts for the progressive spastic paraparesis and atypical cerebral palsy [35]. Thus, ASD in this individual cannot be confidently attributed to SPAST dysfunction alone. Differently, in the other cases, no concurrent genomic alterations were detected, suggesting a potential direct contribution to NDDs (see Table 1).

**Figure 3:**
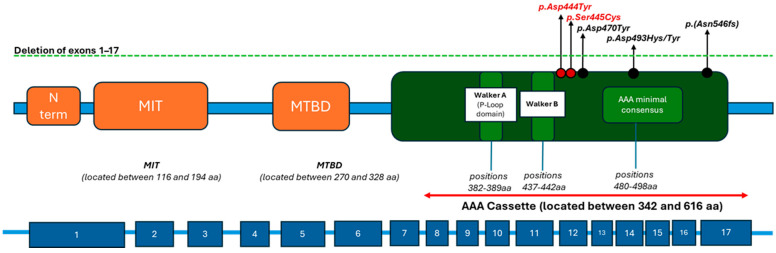
Graphic representation of the spastin protein and its domains. In the orange arrow, patient’s variants, and in *black italics*, all the SPAST defects associated with ASD (green dashed line: deletion of exons 1–17; red circles: variants identified in our patient; black circles: variants reported in patients with *SPAST* and ASD from the literature; below, schematic representation of exons 1–17).

Beyond the phenotypic observations, a closer analysis of the mutational landscape across these five patients suggests a potential functionally critical region within the SPAST gene (see Figure 3). Specifically, four of the five reported variants cluster between c.1300 and c.1650 in the SPAST coding sequence, mapping to the AAA ATPase domain of spastin. The fifth case involves a large deletion encompassing exons 1–17, resulting in complete loss of spastin function and a similarly complex neurodevelopmental phenotype.

This convergence within the AAA domain—a region crucial for spastin’s enzymatic function, including microtubule severing and cytoskeletal regulation—suggests a potential mutational “hotspot” with increased vulnerability. Disruption in this domain could impair both motor pathways and neurodevelopmental processes, such as synaptogenesis and neuronal connectivity [36,37,38].

In our case, two novel de novo missense variants—c.1330G>T (p.Asp444Tyr) and c.1333A>T (p.Ser445Cys)—affect two adjacent and highly conserved residues within the AAA domain. Their spatial proximity, combined with the structural and functional importance of this domain, may raise the hypothesis of an “autism hotspot” within SPAST, where specific variants preferentially impact early cortical wiring and synaptic maturation.

The importance of the AAA domain in regulating microtubule dynamics, neurite outgrowth, and synaptic formation is well established. Disruption of spastin impairs these processes in cellular and animal models. Notably, Lasser et al. demonstrated that many ASD-associated genes converge on microtubule-related pathways, supporting the idea that cytoskeletal dysregulation contributes to the pathophysiology of neurodevelopmental disorders.

The role of spastin in the central nervous system is still not fully elucidated. While it is primarily involved in microtubule severing, it also contributes to cytoskeletal remodeling and calcium homeostasis via store-operated calcium entry (SOCE) [39,40]. It plays a fundamental role in reorganizing microtubule architecture in axons and dendrites—processes essential for establishing proper neuronal connectivity [41,42]. We hypothesize that the complex neurodevelopmental phenotype observed in our patient may result from disrupted SPAST function, leading to altered cortical development and synaptic plasticity. This aligns with recent evidence implicating other microtubule-associated genes in the etiology of neurodevelopmental disorders [38].

Approximately 5% of individuals suspected of a genetic condition, following the application of genome-wide diagnostic approaches, are diagnosed with two or more concurrent variants affecting distinct loci [43], and the chance of a second genetic disorder (rather than an expanded phenotype) should always be taken into account in the presence of complex clinical expressions that do not fit with a single genetic etiology. In our patient, anamnestic, clinical, instrumental, biochemical, and electrophysiological data did not support any other pre-, peri-, or post-natal etiological hypothesis. In addition, array-CGH excluded large chromosomal rearrangements, and whole-exome sequencing (WES) allowed both a deep phenotype-driven targeted analysis—including all known genes associated with developmental disorders, hereditary spastic paraplegia, and metabolic and mitochondrial diseases—and a coverage-based CNV analysis. No additional pathogenic or likely pathogenic variants were identified in other disease-associated genes or genomic regions. However, a dual diagnosis cannot be definitively ruled out, as cryptic variants may escape standard WES analysis. These may include non-coding variants, cryptic splicing alterations, repeat expansions, or mitochondrial DNA variants. Whole-genome sequencing (WGS) represents the gold standard for detecting such variants. Nevertheless, in many countries, including Italy, WGS has not yet been integrated into routine clinical diagnostics due to its higher cost and the increased complexity of laboratory procedures, data analysis, and storage. Until trio-based WGS becomes widely available in clinical practice, periodic re-analysis of WES data (every 1–3 years), in light of evolving scientific knowledge and the patient’s clinical evolution, will be necessary in our child. Similarly, in our child, as in the majority of autistic children, we cannot exclude that ASD may be the consequence of uncharacterized genetic, epigenetic, or environmental factors. Likewise, no history of perinatal hypoxia or neonatal encephalopathy was reported.

In light of the current evidence, the pathogenic role of SPAST in ASD remains uncertain. While a definitive causal relationship cannot be established in the absence of functional validation or large-scale cohort studies, the co-occurrence of ASD and subtle pyramidal signs in patients with SPAST variants—particularly de novo variants affecting the AAA domain—suggests a potential expansion of the phenotypic spectrum. Until more robust evidence is available, we propose that SPAST variants be cautiously considered in the differential diagnosis of early neurodevelopmental disorders when accompanied by subtle motor signs suggestive of pyramidal tract involvement. Future studies, including functional assays and systematic deep-phenotyping, are needed to determine whether SPAST contributes directly to neurodevelopmental phenotypes or acts as a genetic modifier.

Early identification of such features, especially in the context of de novo variants, may enable prompt diagnosis, targeted clinical monitoring, and timely therapeutic interventions. Understanding the genetic mechanisms underlying phenotypic variability, including the role of de novo mutations and their overlap with neurodevelopmental disorders, is essential for refining diagnostic strategies and informing future targeted treatments.

## 5. Conclusions

In conclusion, this case expands the phenotypic and mutational landscape of early-onset and complex SPAST-related HSP by revealing a rare association with severe neurodevelopmental impairment, including ASD. It underscores the importance of considering SPAST variants cautiously in the differential diagnosis of early-onset developmental disorders, even before clear pyramidal signs emerge, and highlights the potential neurodevelopmental impact of mutations affecting the AAA domain of spastin. Additional cases are needed to determine whether ASD represents a coincidental finding in these five patients or may constitute a recurrent phenotypic feature associated with SPAST gene defects.

## Figures and Tables

**Table 1:** Literature overview of SPG4 patients with ASD phenotypes (CTS: corticospinal tract syndrome, PN: peripheral neuropathy, ASD: autism spectrum disorder).

Patients	Spast Mutation	Variant Type	Location (cDNA)	Additional Genomic Variants	SPG4 Form	Motor Phenotype	Neuropsychiatric Phenotype	Spastin Domains
Chelban et al., 2017 [34]	Deletion of exons 1−17	Large deletion	Full Gene Loss	X	Complex	CTS	ASD	AAA Hotspot
Chelban et al., 2017 [34]	c.1408G>T	Missense	c.1408	X	Complex	CTS − PN (motor and sensory)	ASD − memory deficit − seizures − dysphagia	AAA Hotspot
Chelban et al., 2017 [34]	c.1635_1636 insAA	Frameshift	c.1635_1636	X	Complex	CTS	Asperger	AAA Hotspot
Our patient	c.1330G>T and c.1333A>T	De novo missense	c.1330−1333	X	Complex	CTS	Severe ASD	AAA Hotspot

## Data Availability

The original contributions presented in this study are included in the article. Further inquiries can be directed to the corresponding author.
